# Supplementary material for: Neural JNK3 regulates blood flow recovery after hindlimb ischemia in mice via an Egr1/Creb1 axis
Source: Nat Commun. 2019 Sep 17;10:4223. doi: 10.1038/s41467-019-11982-4 (PMC6748991; doi:10.1038/s41467-019-11982-4)
Supplement: Supplementary file 1 — Supplementary information [file 41467_2019_11982_MOESM1_ESM.pdf]

Supplementary information

Neural JNK3 regulates blood flow recovery after hindlimb ischemia in mice via an Egr1/Creb1 axis

Kant et al.

Supplementary Figure1-7

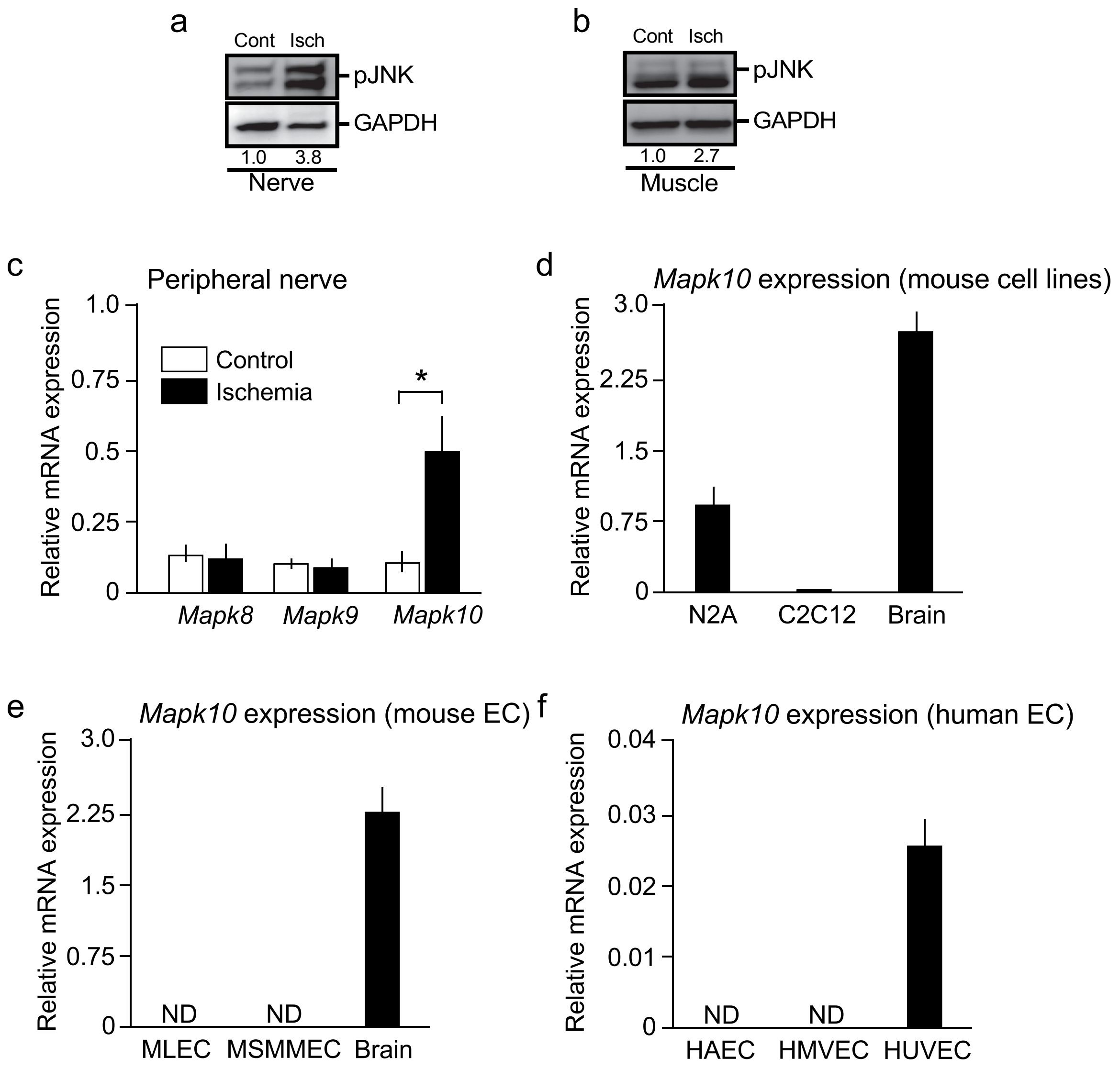

Supplementary Figure 1. JNK phosphorylation and JNK3 expression in different tissues and cell lines. (a-b) Lysate were prepared from human peripheral nerves (a) and muscle (b) and JNK protein phosphorylation were measured by western blot (c) Femoral nerves are isolated from control and hindlimb ischemia leg of mice and qRT-PCR was performed for genes as indicated (n=4 in each group). (d) Neuro-2a (N2a) and C2C12 cell line were exposed to hypoxia for 90 minutes then RNA was isolated and qRT-PCR was performed for *Mapk10* expression (n=3 in each group). (e) RNA was isolated from mouse lung endothelial cells (MLECs) and mouse skeletal muscle microvascular endothelial cells (MSMMECs) and qRT-PCR was performed for *Mapk10* expression (n=3 in each group). (f) RNA was isolated from human aortic endothelial cells (HAECs), Human microvascular endothelial cells (HMVECs), Human umbilical vein endothelial cells (HUVECs) and qRT-PCR was performed for *MAPK10* expression (n=3 in each group). *, $P < 0.05$ by Student's t test. The data are mean ± SEM.

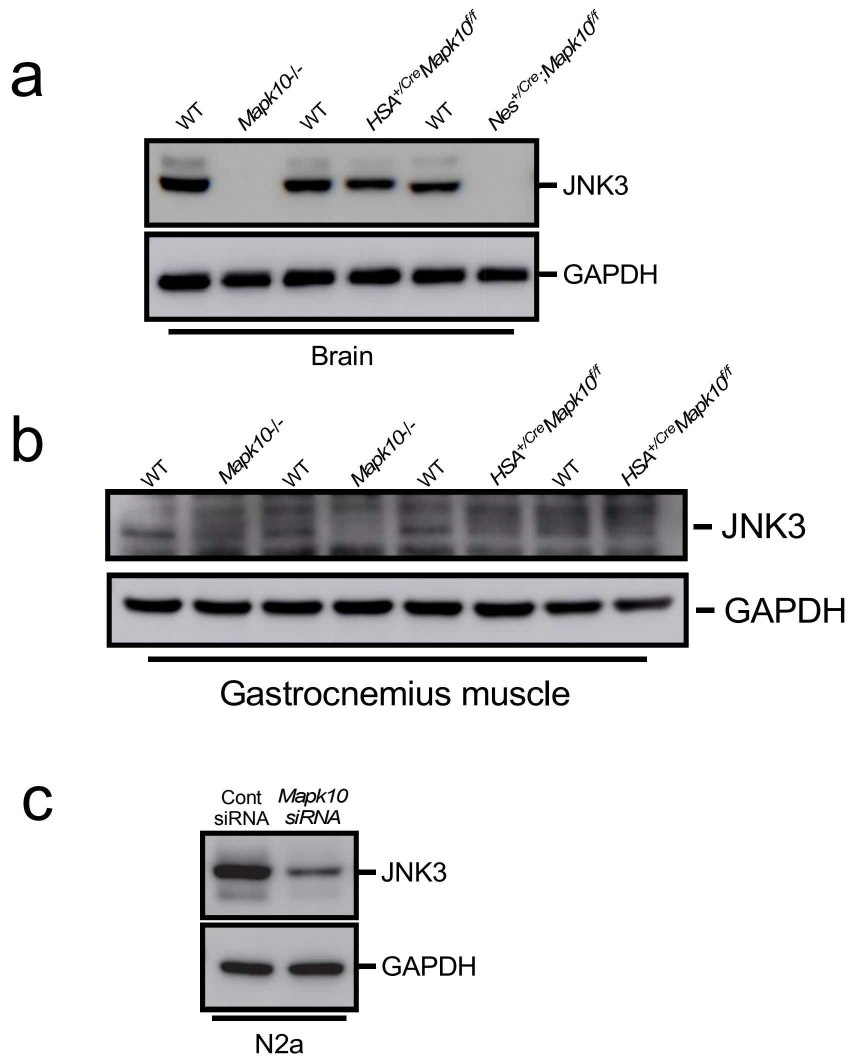

Supplementary Figure 2. JNK3 deficient tissues and cell line. (a) Brain tissues were isolated and immunoblot was performed with antibodies to JNK3 and GAPDH. (b) Gastrocnemius muscle was isolated and immunoblot was performed as described. (c) Extracts prepared from N2a cells either treated with control or *Mapk10* siRNA was examined by immunoblot analysis with antibodies to JNK3 and GAPDH.

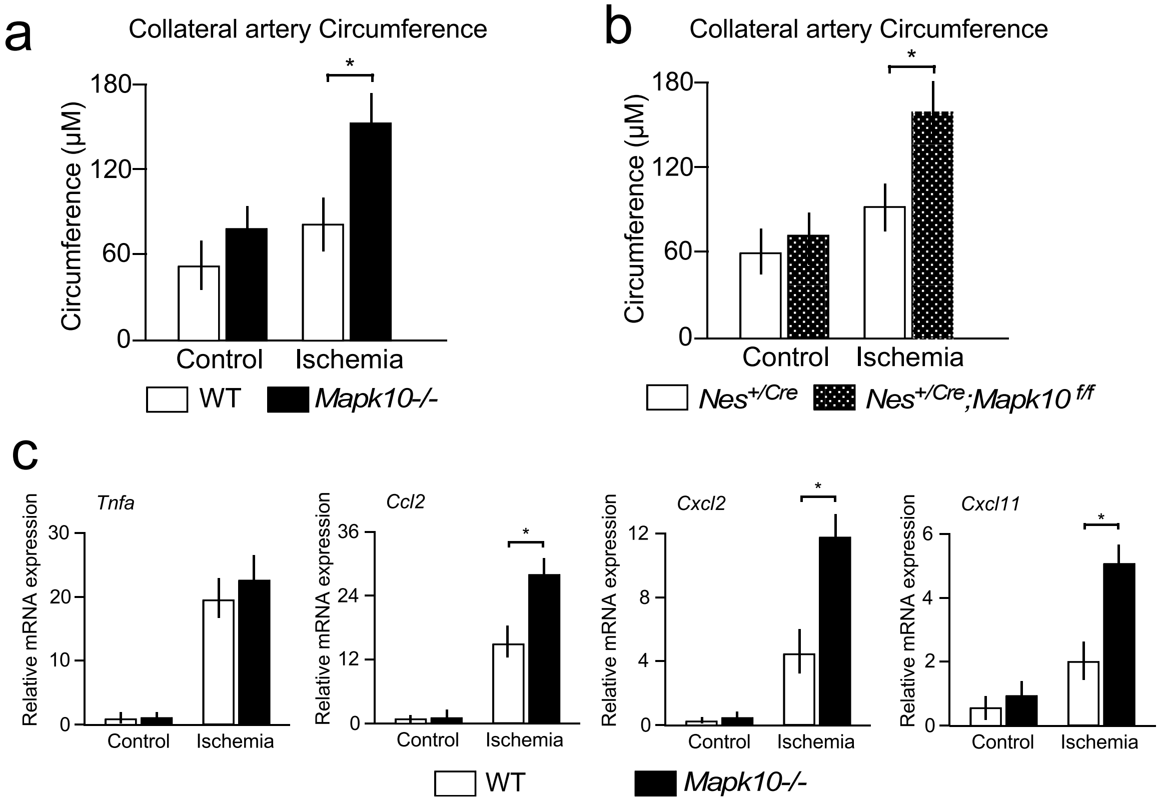

Supplementary Figure 3. Loss of JNK3 promotes collateral arteries circumference and gene expression related to collateral artery remodeling. (a-b) Quantification of circumference of collateral vessels, vWF, in mouse thigh adductor (TA) muscle on Day 21 after ligation of femoral artery in WT and *Mapk10-/-* mice (a) or *Nes*$^{+/Cre}$; *Mapk10*$^{f/f}$ mice (b). (c) qRT-PCR was performed for different genes related to collateral artery remodeling on thigh adductor (TA) muscle from the control and ischemic legs of WT and *Mapk10-/-* mice 3 days post femoral artery ligation (n=3 in each group). Statistically significant differences between groups are indicated (*, $P < 0.05$ by Student's t test). The data are mean ± SEM.

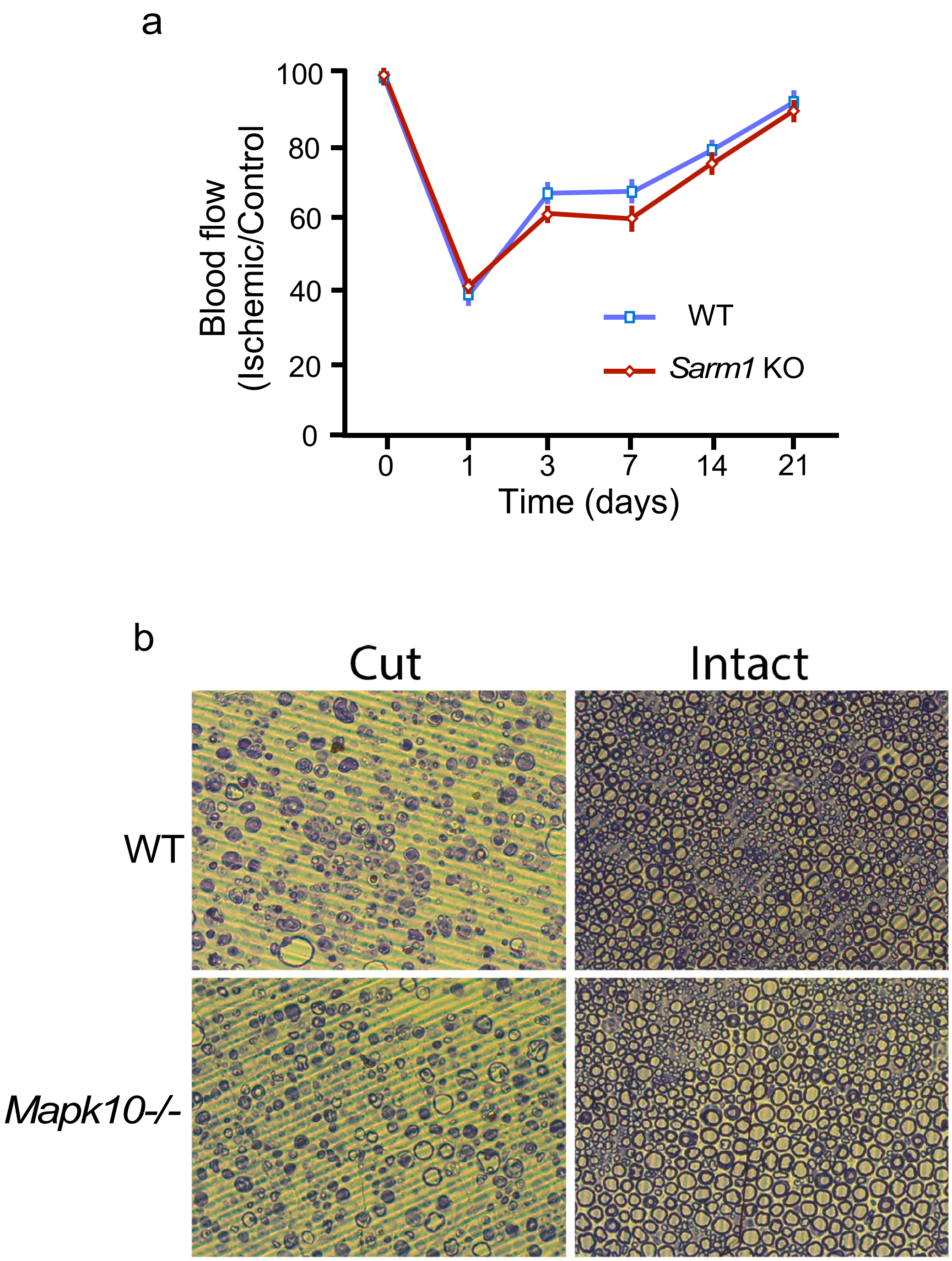

Supplementary Figure 4. Sarm1 knockout and WT blood flow recovery after hind limb ischemia (HLI). (a) Time course of blood flow recovery by Laser Speckle Contrast Imager of WT and *Sarm1-/-* knockout mice (WT, n=7; *Sarm1-/-*, n=5). (b) Representative figures of nerve injury (Cut) or control (Intact) in WT and *Jnk3-/-* mice after axotomy were performed in hindlimb.

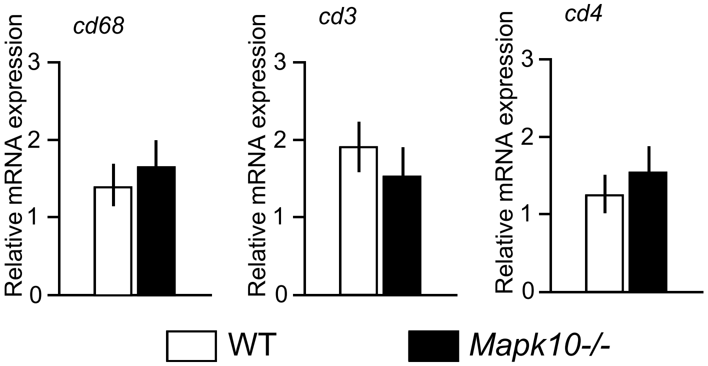

Supplementary Figure 5. Monocytes and T-cells markers expression. RNA was isolated from control and *mapk10-/-* mice after hindlimb ischemia and qRT-PCR was performed for genes as indicated. Statistically significant differences between groups are indicated (n=3 in each group). The data are mean ± SEM.

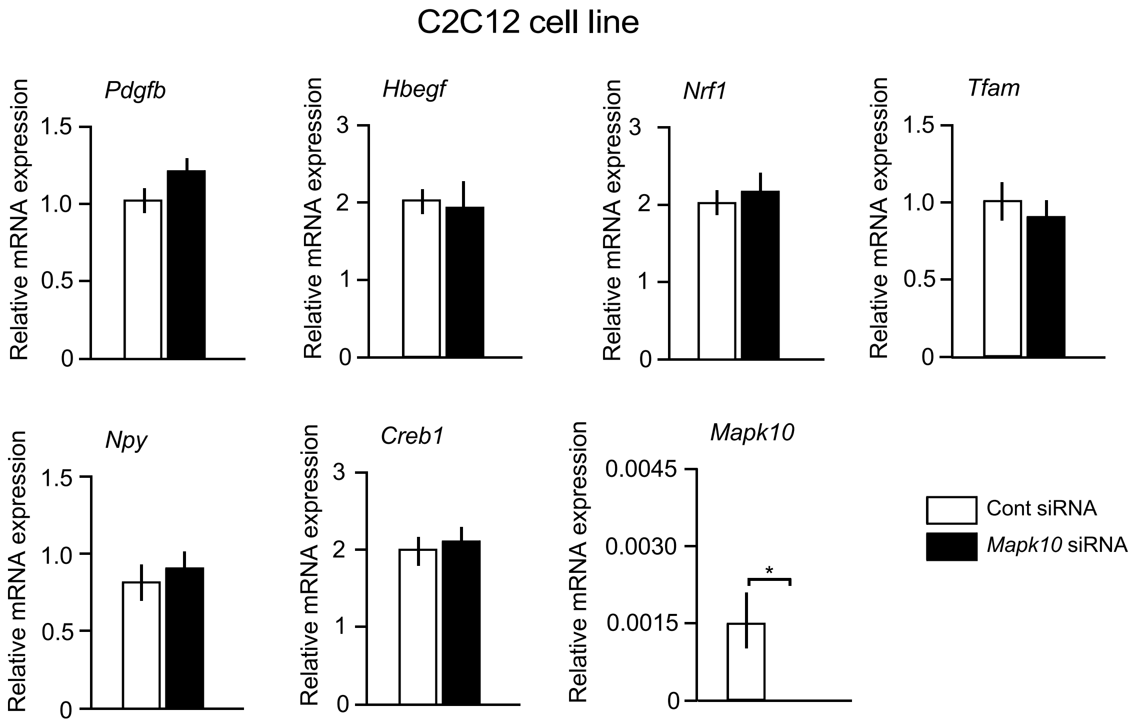

Supplementary Figure 6. Role of JNK3 in C2C12 cell line. (a) Following 90 minutes of hypoxia, RNA was isolated from control and *Mapk10* knockdown C2C12 cells and qRT-PCR was performed for genes as described (n=3 in each group). *, $P < 0.05$ by Student's t test. The data are mean ± SEM.

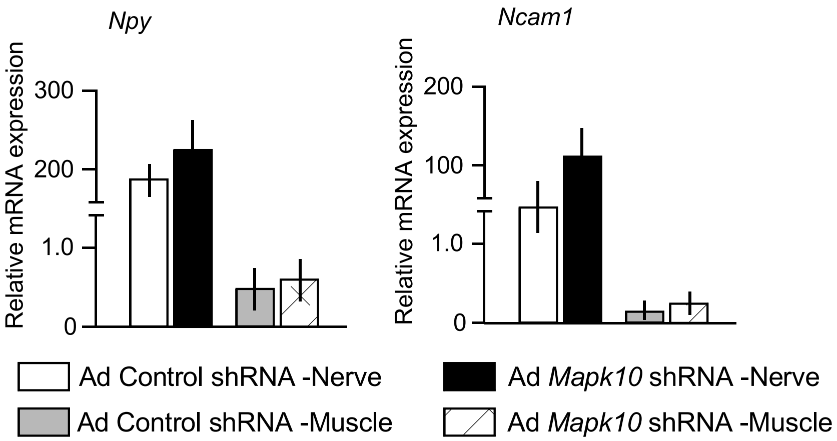

Supplementary Figure 7. Neural gene expression markers. Femoral nerves and muscles tissues were isolated from hindlimb ischemia leg of mice after 48 hours of gene painting with adenovirus expressing control and *Mapk10* siRNA and qRT-PCR was performed for genes as indicated (n=3 in each group).
